# Prognostic Factors in Colorectal Liver Metastases: An Exhaustive Review of the Literature and Future Prospectives

**DOI:** 10.3390/cancers17152539

**Published:** 2025-07-31

**Authors:** Maria Conticchio, Emilie Uldry, Martin Hübner, Antonia Digklia, Montserrat Fraga, Christine Sempoux, Jean Louis Raisaro, David Fuks

**Affiliations:** 1 Faculty of Biology and Medicine, University of Lausanne, 1005 Lausanne, Switzerland; emilie.uldry@chuv.ch (E.U.); martin.hubner@chuv.ch (M.H.); antonia.digklia@chuv.ch (A.D.); montserrat.fraga@chuv.ch (M.F.); christine.sempoux@chuv.ch (C.S.); jeanlouis.raisaro@unil.ch (J.L.R.); david.fuks@chuv.ch (D.F.); 2 Department of Visceral Surgery, Centre Hospitalier Universitaire Vaudois (CHUV), 1005 Lausanne, Switzerland; 3 Department of Oncology, Centre Hospitalier Universitaire Vaudois (CHUV), 1005 Lausanne, Switzerland; 4 Department of Gastroenterology, Centre Hospitalier Universitaire Vaudois (CHUV), 1005 Lausanne, Switzerland; 5 Department of Pathology, Centre Hospitalier Universitaire Vaudois (CHUV), 1005 Lausanne, Switzerland

**Keywords:** colorectal liver metastases, prognostic factors, molecular profiling, precision medicine, artificial intelligence

## Abstract

**Background**: Colorectal liver metastasis (CRLM) represents a major clinical challenge in oncology, affecting 25–50% of colorectal cancer patients and significantly impacting survival. While multimodal therapies—including surgical resection, systemic chemotherapy, and local ablative techniques—have improved outcomes, prognosis remains heterogeneous due to variations in tumor biology, patient factors, and institutional practices. **Methods**: This review synthesizes current evidence on prognostic factors influencing CRLM management, encompassing clinical (e.g., tumor burden, anatomic distribution, timing of metastases), biological (e.g., CEA levels, inflammatory markers), and molecular (e.g., RAS/BRAF mutations, MSI status, HER2 alterations) determinants. **Results**: Key findings highlight the critical role of molecular profiling in guiding therapeutic decisions, with RAS/BRAF mutations predicting resistance to anti-EGFR therapies and MSI-H status indicating potential responsiveness to immunotherapy. Emerging tools like circulating tumor DNA (ctDNA) and radiomics offer promise for dynamic risk stratification and early recurrence detection, while the gut microbiome is increasingly recognized as a modulator of treatment response. **Conclusions**: Despite advancements, challenges persist in standardizing resectability criteria and integrating multidisciplinary approaches. Current guidelines (NCCN, ESMO, ASCO) emphasize personalized strategies but lack granularity in terms of incorporating novel biomarkers. This exhaustive review underscores the imperative for the development of a unified, biomarker-integrated framework to refine CRLM management and improve long-term outcomes.

## Simple Summary

Colorectal liver metastasis (CRLM) represents a major clinical challenge in oncology, affecting 25–50% of colorectal cancer patients and significantly impacting survival. This review synthesizes current evidence on prognostic factors influencing CRLM management, encompassing clinical (e.g., tumor burden, anatomic distribution, timing of metastases), biological (e.g., CEA levels, inflammatory markers), and molecular (e.g., RAS/BRAF mutations, MSI status, HER2 alterations) determinants. The treatment paradigm for CRLM is inherently multidisciplinary, integrating surgical resection, systemic chemotherapy (e.g., FOLFOX, FOLFIRI), and local ablative therapies. Despite the recent advances, clinical decision-making remains complex. This landscape underscores the need for standardized yet personalized approaches. Emerging tools like artificial intelligence (AI) could bridge these gaps by synthesizing molecular data, imaging, and treatment outcomes into predictive algorithms. While AI has shown promise in other malignancies, its application to CRLM remains nascent, representing an untapped opportunity to optimize therapeutic sequencing.

## 1. Introduction

Colorectal cancer (CRC) is the third most prevalent malignancy and the second leading cause of cancer-related mortality worldwide [1]. Despite gradual improvements in survival over recent decades, driven by advances in screening, systemic therapies, and surgical techniques, approximately 25–50% of patients develop colorectal liver metastasis (CRLM), the most common means of metastatic spread [2]. CRLM presents a significant clinical challenge: untreated, median survival rarely exceeds 12–24 months [3]. However, with multimodal management, including liver-directed therapies and modern systemic treatments, long-term survival is now achievable, with 5-year survival rates reaching 71% after R0 resection in selected patients [4].

The treatment paradigm for CRLM is inherently multidisciplinary, integrating surgical resection, systemic chemotherapy (e.g., FOLFOX, FOLFIRI), and local ablative therapies. Surgical resection remains the cornerstone of curative-intent treatment, yet only 20–30% of patients are initially eligible due to anatomical constraints, inadequate future liver remnant, or extrahepatic disease. Innovations such as portal vein embolization, two-stage hepatectomy, and ALPPS have expanded resectability criteria, while non-surgical options (e.g., ablation, Y90 radioembolization) offer alternatives for both resectable and unresectable cases [5].

Despite these advances, clinical decision-making remains complex. Heterogeneity in tumor biology (e.g., RAS/BRAF mutations), patient comorbidities, and institutional preferences often leads to inconsistent treatment strategies. While consensus guidelines (e.g., NCCN, ESMO) provide frameworks for chemotherapy and resection, they lack granularity in terms of integrating molecular profiling and dynamic response assessment [6]. Moreover, disparities persist in defining resectability, with medical oncologists and surgeons frequently diverging in their assessments [7,8].

This landscape underscores the need for standardized yet personalized approaches. Emerging tools like artificial intelligence (AI) could bridge these gaps by synthesizing molecular data, imaging, and treatment outcomes into predictive algorithms. While AI has shown promise in other malignancies, its application to CRLM remains nascent, representing an untapped opportunity to optimize therapeutic sequencing [9].

This exhaustive review examines current evidence and guidelines for CRLM management (NCCN, ESMO, ASCO), highlighting critical gaps in consensus, particularly the underutilization of molecular profiling and the absence of unified decision-making protocols.

## 2. Materials and Methods

This narrative review was conducted to synthesize and critically appraise current evidence regarding the multidisciplinary management of colorectal liver metastases (CRLM), with emphasis on surgical strategies, systemic therapies, molecular profiling, and the evolving role of artificial intelligence (AI) in clinical decision-making.

A purposeful literature search was performed using PubMed, MEDLINE, and Scopus databases, covering publications from January 2000 to April 2025. Search terms included combinations of *“colorectal liver metastases,” “CRLM,” “surgical resection,” “chemotherapy,” “molecular profiling,” “resectability criteria,” “artificial intelligence,” “multidisciplinary care,”* and *“guideline adherence.”* Only articles published in English were considered. Preference was given to high-quality clinical trials, systematic reviews, meta-analyses, international guidelines (e.g., NCCN, ESMO, ASCO), and expert consensus statements. Selected studies were evaluated for relevance based on title and abstract, followed by full-text assessment.

Articles were included if they addressed one or more of the following topics:

- Evidence-based treatment modalities for CRLM (surgery, chemotherapy, local therapies);
- Guideline recommendations for resectability and sequencing of therapies;
- Role of molecular markers (e.g., KRAS, NRAS, BRAF, MSI) in clinical decision-making;
- Use of decision-support tools or AI in managing CRLM;
- Barriers to standardized care in real-world settings.

Exclusion criteria included single-case reports, non-peer-reviewed materials, and articles unrelated to CRLM or without clinical relevance to management decision-making.

Each study was appraised for methodological rigor, significance, and contribution to the overarching clinical framework. Particular attention was paid to areas of controversy or divergence among guidelines, variation in surgical indications, and discrepancies in resectability assessments between disciplines. Evidence was narratively synthesized, with emphasis on

- Consistency or conflict among guidelines;
- Gaps in integrating molecular and imaging data into treatment algorithms;
- Emerging evidence on the role of AI or predictive tools;
- Implications for future clinical pathways and research.

Through this structured narrative approach, we aim not only to consolidate current knowledge but also to highlight unresolved questions, areas of consensus deficiency, and the potential for innovation in CRLM management.

## 3. Results

This section examines factors influencing prognosis in CRLM.

### 3.1. Clinical Prognostic Factors

- Patient demographic characteristics

**Figure 1:**
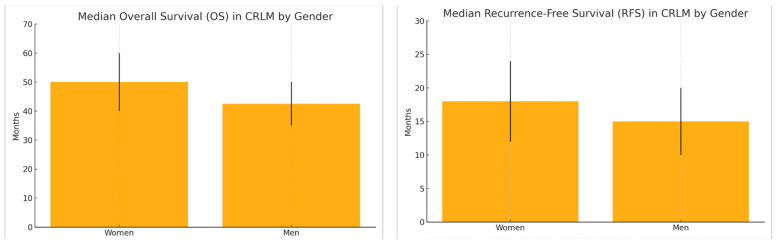
The impact of gender on the overall survival (OS) and recurrence-free survival (RFS).

Gender-based differences significantly impact the management and outcomes of CRLM. While surgical resection remains the cornerstone of curative treatment for CRLM, women are notably 23% less frequently treated with surgery than men, even after accounting for patient and tumor characteristics [10]. This underrepresentation in surgical treatment may help explain observed differences in survival [11]. Women are also more likely to present with right-sided primary tumors and BRAF mutations, both associated with poorer prognosis. Nonetheless, hormonal factors, particularly the potential protective role of estrogen, may contribute to better survival outcomes in women [12]. In terms of chemotherapy, gender influences both tolerability and efficacy. Women are more prone to neurotoxicity from oxaliplatin and to hand–foot syndrome from capecitabine, often requiring dose adjustments that can affect therapeutic efficacy [13]. However, women typically present with fewer comorbidities than men, which may contribute to better systemic treatment tolerance and allow for more optimal dosing. While response rates are generally similar between sexes, women may derive greater benefit from targeted therapies, such as anti-EGFR agents, likely due to differences in tumor biology [14]. Overall, women with CRLM demonstrate better OS and RFS values than men. The median OS ranges from 40 to 60 months in women versus 35 to 50 months in men, while the RFS is typically 12 to 24 months in women compared to 10 to 20 months in men. These differences may be attributable to a combination of biological, hormonal, and treatment-related factors [15] (Figure 1).

Age significantly influences the management of CRLM, impacting decisions regarding surgery, chemotherapy, local ablation, and survival outcomes. In most studies and guidelines, individuals aged 65 and older are considered “elderly”, a definition endorsed by the WHO. Some of the literature further stratifies this population into “young-old” (65–74 years), “middle-old” (75–84 years), and “old-old” (≥85 years), although these distinctions are less relevant in routine oncologic decision-making [16]. Chronological age alone is insufficient to guide treatment choices. Comorbidities, functional reserve, and biological age often better reflect a patient’s ability to tolerate therapies. Elderly patients typically present with higher comorbidity burdens and reduced physiological reserve, warranting a tailored approach. Tools like the G8 or comprehensive geriatric assessment are recommended to assess frailty and guide decision-making [17]. Individualized treatment strategies that weigh therapeutic benefit against potential harm are critical, especially when quality of life is a major concern. Several studies have focused on treatment options in this population. The PANDA trial evaluates the efficacy of panitumumab combined with FOLFOX in patients over 70 years with RAS/BRAF wild-type metastatic colorectal cancer [18]. Importantly, age alone should not exclude patients from curative-intent treatments. Although elderly patients are offered surgery less often, growing evidence shows that, when appropriately selected, they can achieve outcomes comparable to younger individuals [19]. Perioperative complications, such as infections or cardiovascular events, may occur more frequently but can often be mitigated with adequate preoperative optimization [19].

Surgical outcomes in high-volume centers are encouraging. Liver resections in elderly patients do not significantly differ in 30-day mortality compared to younger cohorts. Five-year OS rates reach 30–40 % in selected elderly patients. For younger patients (<70 years), median OS after liver resection ranges from 40 to 60 months, while fit elderly patients (≥70 years) achieve OS values between 30 and 50 months. Recurrence-free survival (RFS) also shows a slight age-related decline, ranging from 12 to 24 months in younger patients and 10 to 20 months in older individuals, likely due to the lower use of adjuvant therapy [20,21]. Systemic chemotherapy remains a key component of treatment in elderly patients, although it is more commonly administered at reduced intensity. Toxicities such as oxaliplatin-induced neuropathy or myelosuppression are more frequent, prompting the use of monotherapy (e.g., 5-FU or capecitabine) or doublet regimens (e.g., FOLFOX or CAPOX) [22]. Despite receiving less combination therapy, response rates in elderly patients remain comparable to those observed in younger cohorts. However, the OS benefit may be somewhat attenuated, likely reflecting the cautious treatment strategies often adopted in this population. For patients unfit for surgery, local ablation techniques, including radiofrequency ablation, microwave ablation, or cryoablation, offer effective alternatives [23,24]. These minimally invasive procedures are particularly suitable for elderly patients with small, unresectable lesions (<3 cm), providing good local control, shorter recovery times, and fewer complications. Although recurrence rates are higher than after surgery, survival benefits remain significant in well-selected patients [25]. Recent guidelines from ESMO, NCCN, and ASCO converge on the importance of personalized treatment in elderly adults with CRLM [26]. They emphasize comprehensive geriatric assessment and advise against using age as the sole criterion to exclude patients from surgery or systemic therapy. Shared decision-making remains essential, particularly when balancing survival with quality of life. In conclusion, while age influences treatment planning, it should not constitute a barrier. With appropriate geriatric assessment, patient selection, and individualized treatment, elderly patients can safely benefit from aggressive and potentially curative therapies.

The ASA physical status classification helps estimate perioperative risk by assessing preoperative health. An ASA score ≥3 indicates significant comorbidities associated with higher complication rates, prolonged recovery, and the need for adjusted treatment plans [27]. This evaluation is essential in multidisciplinary decision-making to balance oncologic benefit and procedural safety. A comprehensive MDT approach, including surgeons, oncologists, hepatologists, and anesthesiologists, is recommended to assess surgical eligibility. Preoperative evaluation should systematically consider cardiovascular, pulmonary, and renal conditions, along with frailty and functional status, which are often more predictive of outcomes than age alone [28]. After initial assessment, these factors can be collectively referred to as major comorbidities requiring careful optimization. While an ASA score ≥3 does not preclude liver resection, it remains a valuable tool to guide multidisciplinary decisions regarding surgical approach, extent of resection, and perioperative care [29]. Indeed, ASA ≥ 3 patients may benefit from preoperative risk mitigation and tailored adjuvant therapies to improve outcomes [30]. These high-risk patients may benefit from targeted preoperative optimization, such as cardiopulmonary assessment, physiotherapy and glycemic control, and from tailored perioperative protocols. Minimally invasive approaches, including laparoscopic and robotic liver resections, are preferred in this context due to their lower complication rates [31]. Enhanced recovery after surgery (ERAS) programs, built on standardized elements like preoperative counseling, optimized pain control and early mobilization, further reducing morbidity and hospital length of stay. In high-risk patients, parenchymal-sparing strategies can be particularly valuable in order to preserve liver function and minimize postoperative morbidity [32].

For patients who are not candidates for surgery, locoregional therapies such as radiofrequency ablation and microwave ablation remain valuable alternatives [33]. However, an ASA score ≥ III may limit feasibility due to comorbidities like cardiac instability or impaired pulmonary function [34]. In selected cases, transarterial chemoembolization and selective internal radiation therapy can serve as palliative options for liver-dominant disease when systemic therapy alone proves insufficient. Regarding chemotherapy, higher ASA scores are associated with reduced tolerance to intensive regimens such as FOLFOXIRI, and a greater risk of toxicity [35]. As a result, treatment is often adjusted with dose reductions or simplified protocols like capecitabine monotherapy in frail patients. ASA status is therefore a useful tool for tailoring treatment intensity to balance efficacy with safety.

b. Number and size of liver metastases

The *number of lesions* in CRLM patients serves as a critical prognostic factor, directly influencing therapeutic options and long-term outcomes. Patients with 1–3 metastases typically experience favorable survival after resection, with 5-year OS rates ranging from 50% to 60% [36]. In contrast, those with ≥4 lesions face greater biological and technical challenges, necessitating multimodal strategies to optimize results. For instance, in the CELIM study, median disease-free survival (DFS) decreased from 16.8 months in patients with fewer than five metastases to 2.5 months in those with more than 10 metastases [37].

For low-burden disease (1–3 lesions), R0 resection is achievable in approximately 80% of cases through anatomic or parenchymal-sparing approaches [38]. Laparoscopic resection is increasingly favored for peripheral lesions (<3 cm). In high-burden disease (≥4 lesions), two-stage hepatectomy with portal vein embolization expands resectability, achieving R0 rates of 50–60% [39]. The ALPPS procedure, considered for rapid liver hypertrophy, carries higher morbidity (~40%). Parenchymal-sparing resections are prioritized to preserve function without compromising oncologic outcomes [40]. Neoadjuvant chemotherapy with regimens such as FOLFOX or FOLFIRI, combined with targeted agents like bevacizumab or cetuximab, can downsize tumors in 40% of cases, enabling resection in 30% of initially unresectable patients [41]. Bevacizumab improves response but requires a 6-week washout period before surgery [42]. Anti-EGFR agents (cetuximab/panitumumab) boost resectability in RAS wild-type tumors. Adjuvant therapy reduces recurrence risk, particularly in patients with ≥4 metastases, with CAPOX or FOLFOX commonly used for 6 months post-resection [43]. Locoregional therapies offer alternative treatment options to surgery for lesions with specific features, but they may also be used in conjunction with surgery or systemic therapy in a multidisciplinary strategy to achieve local control and preserve hepatic parenchyma. Ablation techniques such as radiofrequency ablation and microwave ablation are effective for lesions < 3 cm, with 70% local control and a median OS of 30 months [44]. Transarterial radioembolization and transarterial chemoembolization have response rates of 50%, with a median OS of 24 months in chemo-refractory cases. These therapies are often combined with systemic chemotherapy for a synergistic effect [45].

The *size of lesions* is a critical determinant of prognosis and therapeutic decision-making. Patients with smaller lesions (<5 cm) consistently demonstrate superior outcomes, including a median OS of 60 months compared to 40 months for larger tumors, and 5-year survival rates of 50% versus 30% for lesions ≥5 cm [46]. Additionally, smaller tumors are associated with longer disease-free survival (24 vs. 12 months). Larger metastases correlate with higher recurrence rates, both intrahepatic and systemic, reflecting more aggressive tumor biology [47]. A study from Iran reported a median OS of 43 months for patients undergoing liver metastasectomy, with 1-, 3-, and 5-year survival rates of 91%, 56%, and 42%, respectively. Factors such as tumor size >6 cm, major hepatectomy, rectal primary tumor site, and involved margin <1 mm were associated with decreased OS [48].

For small lesions (<5 cm), R0 resection is achievable in 80% of cases, often with straightforward anatomic or parenchymal-sparing approaches [49]. These procedures are associated with lower postoperative morbidity and excellent long-term survival. In contrast, large CRLM (≥5 cm) require advanced techniques such as two-stage hepatectomy, portal vein embolization, or ALPPS to optimize resectability. Even with successful resection, the median OS remains 30–36 months, underscoring the need for adjuvant strategies [50]. Neoadjuvant chemotherapy (FOLFOX/FOLFIRI ± biologics) can downstage 30–40% of initially unresectable large CRLM, enabling surgery [51]. Bevacizumab improves responses but requires a 6-week washout pre-surgery. Anti-EGFR agents (cetuximab/panitumumab) enhance outcomes in KRAS wild-type tumors. Adjuvant chemotherapy reduces recurrence risk, particularly in large CRLM, with FOLFOX preferred [52]. Ablation techniques such as radiofrequency ablation or microwave ablation offer effective treatment for lesions < 3 cm, with 70% local control and median OS of 30 months. Transarterial radioembolization (TARE) or chemoembolization provides response rates of 50% and median OS of 24 months in chemo-refractory cases [53].

c. Anatomic distribution (unilateral/bilateral)

The anatomical distribution of CRLM (unilateral versus bilateral) significantly influences prognosis and treatment strategies. Unilateral CRLM, accounting for approximately 40–50% of cases, predominantly affects the right hepatic lobe due to its dominant portal blood flow [54]. Surgical resection in these cases yields a 5-year OS rate exceeding 50%, with recurrence rates lower than those observed in bilateral disease [55]. Conversely, bilateral CRLM, more common in synchronous presentations, is associated with higher tumor burden and increased risk of intrahepatic recurrence, leading to shorter DFS even after resection [56]. For unilateral CRLM, standard lobectomy or segmentectomy achieves R0 resection rates above 80%, with durable survival outcomes. Parenchymal-sparing resections are preferred when feasible to preserve liver function. In bilateral CRLM, two-stage hepatectomy, including techniques like ALPPS or portal vein embolization, is employed to maximize resectability [57]. Despite technical challenges, R0 resection is attainable in approximately 60% of selected cases. Notably, a propensity score-matched analysis revealed no significant difference in 5-year OS between patients with bilobar and unilobar CRLM undergoing resection, with rates of 46% and 49%, respectively [58,59].

Neoadjuvant chemotherapy with regimens such as FOLFOX or FOLFIRI, combined with targeted agents like bevacizumab or cetuximab, can convert 30–40% of initially unresectable cases to operable status. Locoregional therapies, including ablation and transarterial chemoembolization, offer a median OS of 24–30 months in chemo-refractory cases [60,61]. A study reported a 5-year OS rate of 44.6% among patients who underwent liver resection after portal vein embolization [62] (Table 1).

d. Extrahepatic metastases

*Pulmonary metastases*

Pulmonary involvement is the most frequent extrahepatic metastatic site in CRLM. The number and size of lung metastases are key prognostic indicators. Patients with fewer than three lesions show better outcomes and are often candidates for curative-intent surgery, whereas a higher number of nodules suggests systemic dissemination and poorer prognosis. Patients with a solitary lung metastasis have a 5-year OS rate of approximately 71%; patients with multiple lung metastases (≥2) exhibit a significantly lower 5-year OS rate of around 48% [63]. Lesions larger than 2–3 cm are associated with decreased survival and higher lymphatic spread, while small nodules (<1 cm) may indicate early dissemination; however, numerous subcentimeter lesions can still represent aggressive disease. Surgical resection remains feasible in selected patients, although when more than ten nodules are present, R0 resection becomes unlikely [64]. Unilateral pulmonary disease offers better prognostic prospects than bilateral metastases. Synchronous metastases (diagnosed within six months of the primary tumor) are associated with worse survival (around 17.6 months) compared to metachronous lesions (18.5 months approximately) [65]. Lymph node involvement, particularly in the hilar or mediastinal areas, markedly worsens prognosis and often precludes surgery [66,67]. Deep-seated lesions, located more than 2 cm from the pleural surface, are technically challenging and associated with higher local recurrence rates, often requiring alternative approaches like stereotactic body radiotherapy (SBRT). In contrast, peripheral lesions are amenable to minimally invasive wedge resections. The treatment strategy involves individualized sequencing of surgery, systemic chemotherapy (e.g., FOLFOX or FOLFIRI ± targeted therapies), and local ablation when surgery is contraindicated [68]. Favorable outcomes are observed in cases of solitary, peripherally located, and metachronous metastases without lymph node involvement, with 5-year survival rates ranging from 30% to 50% following combined hepatic and pulmonary metastasectomy [69].

*Peritoneal metastases*

The management of concomitant CRLM and peritoneal metastases is complex, relying heavily on the Peritoneal Cancer Index (PCI). Patients with PCI < 10 achieve the best outcomes (a 5-year survival rate of 53%), while PCI scores between 10 and 20 are associated with a 5-year survival rate of 23%. This conditions may still allow for cytoreductive surgery combined with hyperthermic intraperitoneal chemotherapy (HIPEC) [70]. A PCI > 20 is generally associated with poor prognosis (5-year survival rate of 12%) and limited surgical benefit [71]. Complete macroscopic resection (CC-0/1) is mandatory for curative intent, and resectable liver metastases do not contraindicate CRS+HIPEC when peritoneal disease is manageable, as shown by Elias et al. [72]. Landmark studies (Verwaal et al., Quenet et al.) established the superiority of cytoreductive surgery HIPEC over chemotherapy alone in selected patients. For borderline-resectable cases, neoadjuvant chemotherapy (FOLFOX/FOLFIRI ± bevacizumab) can enable downstaging [73]. Optimal outcomes are achieved when a low or intermediate PCI is combined with controlled hepatic disease, while systemic therapy remains central in high-PCI cases. Ongoing research focuses on refining patient selection and surgical indications [74,75].

*Bone metastases*

Bone metastases, although less frequent, present unique challenges in CRLM. Prognosis depends heavily on metastatic burden. Patients with oligometastatic bone disease (1–3 lesions) benefit from aggressive local therapy, with 2-year survival rates of 35–50% following surgical resection or SBRT, particularly for lesions compromising mechanical stability or neurological function [76,77]. Diffuse bone involvement typically mandates systemic therapy (FOLFOX/FOLFIRI ± bevacizumab), yielding a median OS of 12–18 months [78]. Bone-modifying agents (bisphosphonates, denosumab) reduce skeletal events by 40%. The Mirels score guides prophylactic stabilization, improving 1-year mobility by 60% in high-risk fractures [79]. Combining local control and systemic therapy improves 5-year survival to 28% in oligometastatic settings, compared to less than 10% in diffuse disease [80]. Emerging data suggest that a subset of oligometastatic patients may achieve 3-year survival with optimized combinatorial strategies, although further validation is needed [81].

*Brain metastases*

Brain metastases indicate a highly aggressive disease course in CRLM. Three factors guide management: metastatic burden, lesion characteristics, and patient fitness. Oligometastatic brain disease (1–3 lesions) treated with local therapies achieves a median OS of 8–12 months [82]. Stereotactic radiosurgery (SRS) provides 1-year local control rates of 70–80%, while surgical resection improves survival to 10–14 months in solitary lesions [83]. For larger (>3 cm) symptomatic metastases, surgery reduces mortality from the mass effect by 50% compared to SRS alone [84]. Deep-seated or eloquent-area lesions favor SRS, achieving 1-year progression-free survival of 60% [85]. Multifocal disease often requires whole-brain radiotherapy (WBRT), associated with a median OS of 4–6 months and significant neurocognitive decline (30–40%) [86]. Systemic therapies have limited efficacy; regorafenib achieves intracranial response rates of only 5–15%. Multidisciplinary management combining local therapies and systemic strategies leads to 2-year survival rates of 20–25% in carefully selected patients [87,88].

e. Characteristics of primary tumor

The histological features of primary colorectal tumors play a critical role in determining prognosis and guiding treatment strategies for CRLM. Key factors such as lymph node involvement, tumor differentiation, vascular emboli, perineural invasion, and microsatellite instability (MSI) status influence tumor behavior and therapeutic response [89,90]. *Lymph node positivity* is associated with more aggressive disease, correlating with higher rates of liver metastases and poorer survival outcomes. Patients with lymph node-positive tumors have a 5-year survival rate of approximately 18.1%, compared to 23.2% for node-negative cases, justifying the use of aggressive systemic therapies, including adjuvant chemotherapy [91]. *Tumor differentiation* also significantly impacts prognosis. Well-differentiated (G1) tumors exhibit a 5-year survival rate of 68.9%, whereas poorly differentiated (G3) tumors show a sharp decline to 39.5%. Poor differentiation often warrants consideration of neoadjuvant chemotherapy prior to surgery, whereas surgery alone may suffice for well-differentiated tumors in the absence of other high-risk features [92]. Histological markers such as *vascular emboli* and *perineural invasion* indicate a higher likelihood of hematogenous dissemination and recurrence, supporting early initiation of systemic therapy and intensified postoperative surveillance [93].

*Microsatellite instability* (MSI) status represents a distinct prognostic and therapeutic dimension. In early-stage colorectal cancer, high-MSI (MSI-H) tumors are associated with a favorable prognosis. However, in metastatic settings, MSI-H tumors often show resistance to standard fluoropyrimidine-based chemotherapy while exhibiting exceptional responses to immunotherapy. In patients undergoing metastatectomy, median survival for MSI-H metastatic CRC can reach up to 33.8 months [94].

*Right-sided colon cancer*

Right-sided colon cancer is characterized by distinct molecular features, including higher rates of microsatellite instability, BRAF mutations, and CpG island methylation. These tumors frequently present at an advanced stage, with more aggressive biological behavior and lower responsiveness to systemic therapies, particularly to anti-EGFR agents like cetuximab. Consequently, patients with resected right-sided colon cancer liver metastases exhibit a 5-year OS rate of approximately 27.3% [95]. Although immunotherapy shows promise for MSI-H RSCC, treatment options for MSS right-sided colon cancer remain limited.

*Left-sided colon cancer*

In contrast, left-sided colon cancer more often harbors RAS and BRAF wild-type profiles and displays higher EGFR expression, rendering these tumors more responsive to anti-EGFR therapies. Patients with left-sided colon cancer -derived CRLM tend to achieve better chemotherapy sensitivity, higher resectability rates, and improved long-term outcomes. For resected left-sided colon cancer liver metastases, the 5-year OS rate reaches approximately 51.8% [96]. Complex surgical strategies such as two-stage hepatectomy or ALPPS are more feasible in left-sided colon cancer due to more favorable tumor biology [97].

*Rectal cancer*

Although biologically close to left-sided colon cancer, rectal cancers demonstrate distinct metastatic patterns, with a higher incidence of synchronous liver metastases and a greater propensity for extrahepatic spread [98]. Their pelvic location and frequent need for neoadjuvant chemoradiation require tailored management strategies, including total neoadjuvant therapy or coordinated surgical approaches. Patients with resected rectal cancer and CRLM achieve a 5-year OS rate of approximately 60.8%, though the complexity of pelvic disease often necessitates highly integrated systemic and surgical treatment plans [99].

f. Timing of onset (synchronous vs. metachronous)

Synchronous CRLM are defined as liver metastases identified at the time of the primary colorectal cancer diagnosis or within a short time frame thereafter. Recent studies indicate that the incidence of synchronous CRLM ranges from 13.8% to 17.1%, with approximately 20% of colorectal cancer patients presenting with metastatic disease at diagnosis [100]. Prognostically, the presence of synchronous liver metastases is associated with a poorer outcome compared to metachronous metastases. Five-year survival rates for patients with synchronous CRLM have been reported between 20% and 50%, depending on factors such as the extent of liver involvement and the feasibility of surgical resection [101]. The liver-first approach, where liver metastases are addressed before the primary tumor, has shown promising results, particularly in patients with multiple bilobar metastases, offering improved survival outcomes [102,103].

Primary tumor location directly informs therapeutic strategies. In right-sided colon cancer, VEGF inhibitors (e.g., bevacizumab) are generally favored over anti-EGFR therapies, with immunotherapy prioritized for high-MSI tumors [104]. In left-sided colon cancer, maximizing anti-EGFR efficacy in RAS wild-type cases and pursuing aggressive surgical strategies is often feasible and beneficial. For rectal cancer, a multimodal approach (integrating pelvic and hepatic disease control) is critical to optimizing outcomes [105].

*Asymptomatic colorectal cancer and resectable synchronous CRLM*

In this favorable scenario, preoperative chemotherapy is recommended, as supported by a 91% consensus among experts [106]. Four to six cycles are advised. Data from the LiverMetSurvey show no survival advantage for chemotherapy-first methods compared to surgery-first methods, with 5-year survival rates of 42% versus 47%, respectively [107]. This likely reflects selection bias, as patients undergoing primary tumor resection first had less extensive disease. For mid- and low-intensity rectal cancers, radiotherapy is often necessary, and the simultaneous resection of the primary tumor and metastases should be avoided [108]. For colonic and upper rectal tumors, one-stage surgery is not recommended for complex tumors, high-risk patients, or when major hepatectomy (P3 segments) is planned [105]. Irrespective of the primary tumor status, one-stage resection is associated with a lower 5-year survival (40%) compared to liver-first (47%) or colon-first (44%) strategies [109]. Chemotherapy-first (±radiotherapy) treatment is recommended, followed either by staged surgery or, in selected patients with limited hepatic disease and easily resectable primary tumors, a one-stage procedure [110,111]. However, no strong evidence clearly favors chemotherapy-first treatment over colon-first strategies.

*Asymptomatic colorectal cancer and non-resectable synchronous CRLM*

In this scenario, initial chemotherapy is the consensus method to achieve resectability. Optimal regimens include doublets or triplets plus biologics. Data from the LiverMetSurvey show similar 5-year survival between chemotherapy-first and colectomy-first approaches (31% vs. 33%), although a selection bias likely exists [112,113]. If CRLM becomes resectable, the preferred approach is to prioritize liver resection before primary tumor surgery. According to the LiverMetSurvey, the 5-year survival rate was 42% for liver-first, compared to 33% for colon-first surgery and 28% for one-stage surgery [114,115]. In rectal cancer, short-course radiotherapy followed by chemotherapy, or chemotherapy followed by radiotherapy with resection of CRLM during the interval, are considered valid strategies. Preoperative chemotherapy aimed at sphincter preservation alone is not recommended according to NICE guidelines [116]. In summary, the recommended strategy is to initiate optimal chemotherapy first to achieve liver metastasis resectability, then proceed with hepatic resection followed by primary tumor surgery.

*Symptomatic colorectal cancer and resectable synchronous CRLM*

Patients presenting with symptomatic colorectal cancer such as bleeding, obstruction, or perforation, require tailored management. Bleeding is usually manageable with blood transfusions, and it is recommended to proceed with chemotherapy first, while 25% favor immediate tumor resection [117]. For bowel perforation, surgery is necessary: the resection of the primary tumor is preferred when feasible (right hemicolectomy for right-sided tumors, sigmoid colectomy for sigmoid tumors), while stoma creation is recommended when surgery is technically challenging (e.g., low anterior resection, total mesorectal excision) [118]. In cases of obstruction, stenting is generally discouraged due to poor outcomes and should be reserved for elderly patients with easily resectable lesions; it is not advised for right-sided or rectal tumors, especially when anti-angiogenic therapies are planned [119]. For proven complete occlusion, primary tumor resection is necessary, with the possibility of surgery with stoma or considering stenting when possible according to the position [120]. In summary, for perforated or obstructive tumors, the resection of the primary tumor is indicated first, followed by chemotherapy and liver surgery; for bleeding tumors without obstruction, chemotherapy is the preferred initial approach.

*Symptomatic colorectal cancer and non-resectable synchronous CRLM*

In patients with symptomatic colorectal cancer and non-resectable CRLM, the goal remains to achieve liver metastases resectability. Surgery of the primary tumor should be reserved for cases of bowel perforation or occlusion, while systemic chemotherapy should be initiated in other cases to downsize both the metastases and the primary tumor [121]. The use of stents is generally discouraged given the high risk of complications. In summary, for perforated or occlusive tumors, primary tumor resection should precede chemotherapy, with subsequent liver surgery if downsizing is achieved. For tumors causing anemia due to bleeding, induction chemotherapy should be initiated first to control both primary and metastatic disease, followed by surgery of the dominant tumor burden, typically the liver, through a reverse approach [118,119].

g. Resectability criteria and strategies to optimize surgical eligibility in CRLM

*Definition of Resectability*

Resectability traditionally requires the complete resection of all visible disease tissue with negative microscopic margins (R0), while preserving a sufficient volume of functional liver parenchyma. Typically, a future liver remnant (FLR) of at least 20–30% of the total liver volume is necessary in patients with normal liver function, and higher thresholds (30–40%) are recommended in cases of chemotherapy-induced liver injury or underlying liver disease [122].

Recent concepts extend the definition beyond purely technical feasibility. “Oncological resectability” emphasizes achieving durable disease control, integrating factors such as tumor biology (RAS/BRAF mutation status, response to systemic therapy) and patient’s general condition into surgical decision-making [123,124]. Some centers now consider resection even in cases of bilobar, multifocal metastases, provided complete macroscopic removal is achievable in combination with systemic therapy.

*Anatomical and functional constraints*

Resectability is impacted by the involvement of major vascular and biliary structures. Portal vein infiltration, hepatic vein invasion (especially the three main hepatic veins), and biliary obstruction must be carefully evaluated. For instance, median OS decreases from 60 to 36 months, and recurrence rates more than double, reflecting both the technical difficulty of resection and the tumor’s inherent aggressiveness [125]. Indeed, these metastases often necessitate modified surgical approaches, as conventional hepatectomy techniques risk leaving positive margins or compromising essential vascular patency. Hepatic vein invasion significantly impacts survival, reducing median OS from 60 to 36 months, with 5-year survival dropping from 50% to 30% compared to cases without vein involvement. In selected patients, R0 resection with vein reconstruction can achieve an OS of 30–36 months [126]. Neoadjuvant FOLFOX therapy enables resection in about 30% of cases, with adjuvant therapy improving DFS. Advanced surgical techniques allow for vascular reconstruction when necessary, but they increase complexity and perioperative risk. Biliary involvement similarly lowers OS, reducing median survival to 30–36 months compared to 60 months in non-involved cases. While resection with biliary reconstruction remains feasible, it increases the risk of liver dysfunction [123].

Preservation of adequate liver function is critical. Preoperative liver volumetry, dynamic liver function tests (e.g., indocyanine green retention rate, LiMAx test), and imaging-based assessments are used to the predict postoperative hepatic reserve. Particular attention is needed in patients previously exposed to hepatotoxic chemotherapy (e.g., oxaliplatin, irinotecan) [127].

*Technical strategies to optimize resectability*

- **Portal vein embolization (PVE):** This is the standard method to induce hypertrophy of the future liver remnant. It achieves 10–20% FLR growth within 3–8 weeks. Morbidity is low (~2–5%), and the dropout rate for resection is about 20%. Two-stage hepatectomy (TSH) offers 35–45% 5-year survival rates when completed, though up to 20–30% of patients may experience progression and dropout between stages [128].
- **Liver venous deprivation (LVD):** This combines the embolization of the portal vein and the hepatic vein of the liver to be resected. This leads to more rapid and greater hypertrophy (~40–50% FLR increase within 2–4 weeks) compared to PVE alone. Early data suggest a comparable safety profile [129].
- **Associating liver partition and portal vein ligation for staged hepatectomy (ALPPS):** ALPPS, which induces rapid hypertrophy (~70–80% in 7–10 days), carries a 5–10% mortality risk but offers faster liver regeneration. Minimally invasive resections have reduced morbidity by up to 40%, improving recovery times and patient outcomes. It is indicated mainly in highly selected patients with insufficient FLR and good performance status [130].

*Oncological strategies to optimize resectability*

Triplet regimens (FOLFOXIRI ± targeted agents) improve response rates compared to doublets (FOLFOX, FOLFIRI), offering greater potential for tumor shrinkage. FOLFIRI + Bevacizumab offer a median OS approximately 30.2 months and a median progression free survival of approximately 12.4 months. FOLFIRI + Cetuximab offer a median OS approximately 31.9 months and a median progression free survival of approximately 11.7 months, compared to doublets median OS of approximately 29.8 months and a progression free survival of around 9.7 months. Quadruplet combinations with targeted therapies (e.g., anti-EGFR or anti-VEGF depending on RAS/BRAF status) may further enhance resectability in selected patients [131].

Patients with initially unresectable CRLM may become eligible for resection after effective systemic therapy. Reported secondary resection rates after conversion therapy range from 15 to 40%, depending on disease burden and chemotherapy regimens used [132]. Tumor response, assessed using imaging (RECIST criteria) and biochemical markers (CEA drop), guides the re-evaluation for surgical intervention (Table 2).

### 3.2. Biological Prognostic Factors

- Pre- and postoperative CEA levels

Carcinoembryonic antigen (CEA) remains a key biomarker in the management of CRLM, with well-established prognostic significance. Patients presenting with normal CEA levels (<5 ng/mL) exhibit a median OS of approximately 60 months, compared to 40 months in patients with elevated CEA levels. Furthermore, normal preoperative CEA is associated with a higher likelihood of achieving R0 resections, with a two-fold increase in the probability of obtaining negative microscopic margins during liver surgery [133]. Elevated CEA levels are indicative of a more aggressive disease phenotype and are associated with the need for intensified multimodal therapy. In these patients, neoadjuvant chemotherapy is often required to reduce tumor burden before surgical intervention, with the potential addition of targeted therapies such as bevacizumab or cetuximab in cases with favorable molecular profiles [134]. The postoperative monitoring of CEA is critical, as elevated postoperative levels are predictive of early recurrence [135]. Serial measurements of CEA have become an integral part of treatment planning, guiding both systemic therapy adjustments and surgical strategies. The prognostic value of CEA is further enhanced when combined with emerging biomarkers such as circulating tumor DNA (ctDNA), allowing for a more dynamic assessment of tumor behavior [136,137]. Current research focuses on refining CEA thresholds to improve risk stratification and to further personalize treatment pathways for patients with CRLM [138,139].

b. Inflammatory markers: Neutrophil–Lymphocyte Ratio and PLR The Platelet–Lymphocyte Ratio

Easily calculated from routine blood counts, the neutrophil–lymphocyte ratio and platelet–lymphocyte ratio reflect the interplay between systemic inflammation and immune competence. An elevated preoperative neutrophil–lymphocyte ratio is consistently associated with poorer outcomes in CRLM, including reduced overall and disease-free survival after liver resection [140,141]. Biologically, it reflects a pro-tumor environment, where neutrophils promote progression and lymphopenia weakens immune defense. Postoperative spikes in the neutrophil–lymphocyte ratio also correlate with early recurrence, making it a valuable tool for dynamic monitoring [142,143].

Similarly, an elevated platelet–lymphocyte ratio before surgery predicts worse prognosis, likely due to platelet-derived tumor-promoting factors and impaired lymphocyte-mediated immunity. In MSI-H CRLM, a high platelet–lymphocyte ratio may also suggest reduced responsiveness to immunotherapy [144,145]. Composite scores combining neutrophil–lymphocyte ratio and PLR offer enhanced risk stratification but remain investigational. While not yet endorsed by major guidelines, the accessibility and reproducibility of these biomarkers support their potential integration into future risk-adapted treatment strategies [146]. Larger validation studies and correlations with molecular tools like ctDNA are needed to confirm their role in personalizing CRLM care [147,148].

c. Nutritional and functional reserve: the role of albumin, sarcopenia and nutritional status

The nutritional and metabolic status of patients with CRLM, reflected in biomarkers such as serum albumin and sarcopenia, is a key determinant of systemic inflammation, physiological reserve, and treatment tolerance. These factors significantly influence surgical risk, chemotherapy response, and long-term survival [149]. Serum albumin, synthesized by the liver, reflects both nutritional state and inflammatory burden. Hypoalbuminemia (typically <3.5 g/dL) is associated with impaired hepatic function, chronic catabolism, and altered drug metabolism [150]. Clinically, it correlates with increased postoperative complications (infections, delayed recovery), reduced chemotherapy tolerance (frequent dose reductions), and poorer survival following resection [151].

Sarcopenia, defined by the loss of muscle mass and strength, further amplifies these risks. Although commonly assessed by imaging at the L3 vertebral level, its clinical impact is clear: it is linked to 30–50% higher postoperative complication rates, longer ICU stays, and up to 40% lower progression-free survival during systemic therapy [152]. Five-year OS drops below 45% in sarcopenic patients, compared to over 65% in those with preserved muscle mass [153,154]. These biomarkers also guide clinical action. Preoperatively, nutritional optimization (following ESPEN guidelines) aims to correct hypoalbuminemia through targeted support, while prehabilitation programs combining protein supplementation and resistance training can reverse sarcopenia within 8–12 weeks. During chemotherapy, sarcopenic patients often require dose reductions (e.g., 20% for oxaliplatin), and hypoalbuminemia calls for close metabolic monitoring to prevent toxicity. When both albumin levels are low and sarcopenia is present, a shift toward palliative strategies may be appropriate; conversely, albumin >3.8 g/dL combined with preserved muscle mass supports more aggressive, curative-intent approaches. Emerging pharmacologic strategies, such as ghrelin agonists, and the integration of biomarker-driven decision pathways may further personalize CRLM management [155,156]. For now, the early identification and correction of these modifiable factors remain essential to improve outcomes and tailor care.

d. Pharmacological sensibility

Hypersensitivity reactions to commonly used chemotherapeutic agents (including oxaliplatin, irinotecan, and 5-fluorouracil (5-FU)) are a critical concern in CRLM management. Oxaliplatin hypersensitivity affects approximately 10–20% of patients, often presenting as acute infusion reactions. Irinotecan, though less frequently allergenic, can induce cholinergic syndromes and rare hypersensitivity events. 5-FU reactions are uncommon but potentially severe, typically linked to dihydropyrimidine dehydrogenase deficiency, which compromises drug metabolism and exacerbates toxicity [157]. Targeted therapies also carry immunogenic risks. Cetuximab induces infusion-related reactions in up to 20% of patients, while bevacizumab can cause rare but serious hypersensitivity responses [158,159]. Management strategies, including premedication with corticosteroids and antihistamines or desensitization protocols, enable continuation of these effective treatments despite previous reactions [160]. Pharmacogenetic testing plays an increasing role in tailoring CRLM therapy. Dihydropyrimidine dehydrogenase deficiency, present in 3–5% of the population, reduces clearance of 5-FU and capecitabine, heightening the risk of myelosuppression and mucositis. Routine dihydropyrimidine dehydrogenase deficiency mutation screening and uracil plasma level measurements identify at-risk patients, allowing dose adjustment or alternative regimens like TAS-102 or irinotecan [161,162]. Similarly, UGT1A1 polymorphisms, particularly UGT1A1*28, impair irinotecan metabolism and raise the risk of neutropenia and diarrhea, warranting dose reductions for homozygous carriers. Thymidylate synthase expression levels also modulate 5-FU sensitivity, with high expression predicting reduced efficacy. Emerging biomarkers such as microRNAs regulating thymidylate synthase offer additional predictive potential [163]. A personalized approach integrating allergy risk assessment, pharmacogenetic profiling, and protocol adaptation, is essential for maximizing therapeutic efficacy while minimizing toxicity. These strategies are now supported by major oncology guidelines, reinforcing the central role of individualized medicine in managing metastatic colorectal cancer [164].

### 3.3. Molecular and Histopathological Prognostic Factors

- KRAS, NRAS and BRAF mutations

*KRAS mutations* are present in approximately 35–45% of metastatic colorectal cancer cases, with NRAS mutations occurring in a smaller subset [165]. These mutations are pivotal in the RAS-MAPK signaling pathway, leading to constitutive activation that promotes tumorigenesis. In the context of CRLM, RAS mutations are associated with resistance to anti-EGFR therapies, such as cetuximab and panitumumab. Patients with RAS-mutated CRLM undergoing liver resection exhibit a median OS of approximately 55.3 months, with a 5-year survival rate of 46.6% [166]. In contrast, RAS wild-type patients demonstrate superior outcomes, underscoring the prognostic significance of RAS mutation status in treatment planning.

*BRAF mutations*, particularly the V600E variant, are less common in CRLM but are associated with a more aggressive disease course. The incidence of BRAF mutations in CRLM ranges from 1% to 6.1%, with V600E being the predominant subtype. These mutations are linked to rapid tumor progression and poor prognosis [167]. A multicenter study involving 105 patients with BRAF-mutated CRLM reported a median OS of 16.2 months, with a 1-year survival rate of 65% and a 3-year rate of 16% [168,169]. Notably, surgical resection of both the primary tumor and CRLM significantly improved survival outcomes, highlighting the potential benefit of aggressive surgical intervention in this subset of patients. The presence of KRAS, NRAS, and BRAF mutations necessitates tailored therapeutic strategies. For RAS-mutated tumors, anti-EGFR therapies are generally ineffective; thus, treatment regimens often incorporate anti-VEGF agents like bevacizumab [170,171]. In BRAF-mutated cases, the addition of targeted therapies, such as BRAF inhibitors, in combination with MEK inhibitors, has shown promise in clinical trials [172,173]. In conclusion, the mutational landscape of CRLM profoundly influences therapeutic decisions and prognostication. Comprehensive molecular profiling is essential for optimizing treatment strategies and improving patient outcomes.

b. MSI-H Status

MSI-H tumors exhibit a high mutational burden, leading to increased neoantigen expression and enhanced immunogenicity. This profile renders them particularly responsive to immune checkpoint inhibitors such as pembrolizumab and nivolumab [174]. In CRLM, MSI-H status has been associated with significantly improved OS when treated with immune checkpoint inhibitors. A population-based study utilizing the National Cancer Database reported an adjusted hazard ratio (aHR) of 0.57 (95% CI: 0.43–0.77) for OS in MSI-H patients receiving immunotherapy compared to chemotherapy. In a notable UK-based trial, 32 patients with stage II/III MSI-H colorectal cancer were treated with pembrolizumab prior to surgery. Results indicated that 59% of patients had no detectable cancer post-treatment, suggesting a potential for immunotherapy to obviate the need for surgery and chemotherapy [175,176].

In contrast, MSS tumors, characterized by proficient DNA mismatch repair and lower mutational burdens, demonstrate limited responsiveness to immune checkpoint inhibitors. A study analyzing 21,951 mCRC patients found no significant survival advantage with immunotherapy in the MSS cohort (aHR: 0.94; 95% CI: 0.69–1.29) [177]. Furthermore, the presence of liver metastases in MSS mCRC patients has been associated with resistance to immunotherapy. A cohort study indicated that MSS patients without liver metastases may derive clinical benefits from PD-1/PD-L1 inhibitors, whereas those with CRLM exhibited poor responses [178,179]. Beyond treatment response, MSI status holds prognostic significance in CRLM. A national analysis revealed that patients with MSI tumors undergoing metastasectomy had a median OS of 33.2 months, compared to 41.1 months in MSS patients [180,181]. This finding underscores the complex interplay between molecular characteristics and clinical outcomes in CRLM (Figure S1 in Supplementary Materials).

c. HER2/neu Alterations

Although HER2 is a well-established oncogene in breast and gastric cancers, it has recently gained recognition as a clinically significant biomarker in approximately 3–5% of CRLM, particularly among KRAS/NRAS wild-type tumors [182]. This molecular subset exhibits distinct biological behavior, often associated with left-sided primary tumors and a more aggressive clinical course. HER2 amplification (detected in 2–5% of cases) confers innate resistance to anti-EGFR therapies, even in otherwise eligible patients, underscoring its dual prognostic and therapeutic relevance [183,184].

Recent advances in targeted therapies have shown encouraging results. Dual HER2 blockade strategies, such as trastuzumab combined with pertuzumab, have demonstrated response rates ranging from 30% to 50% [185,186]. Furthermore, newer antibody-drug conjugates, such as trastuzumab deruxtecan, are showing expanding potential in early-phase studies [187]. Nonetheless, resistance mechanisms (particularly involving co-alterations in PIK3CA or PTEN) highlight the importance of comprehensive molecular profiling to guide treatment selection.

Reflecting this paradigm shift, both ESMO and NCCN guidelines now recommend routine HER2 testing in RAS wild-type mCRC, particularly for left-sided tumors, establishing HER2 as a clinically actionable target within contemporary precision oncology strategies [188,189].

d. POLE, POLD1 and PD-L1

Mutations in the exonuclease domains of the POLE and POLD1 genes (present in ~1–2% and <1% of colorectal cancers, respectively) lead to a hypermutated phenotype characterized by defective DNA proofreading [190]. These alterations are associated with high tumor mutational burden and a notably favorable prognosis, even in microsatellite-stable (MSS) tumors. In CRLM, POLE-mutated patients demonstrate robust responses to immune checkpoint inhibitors, with outcomes comparable to those seen in MSI-H tumors. Similar trends are emerging for POLD1 mutations, though data remain more limited [191]. These genomic alterations also appear to enhance sensitivity to platinum-based chemotherapy and may co-occur with other actionable mutations, supporting their inclusion in comprehensive molecular profiling.

In parallel, the immune microenvironment of CRLM reveals unique PD-L1 dynamics, with metastatic lesions often expressing higher levels than their primary tumor counterparts, likely reflecting liver-specific immune editing. In MSI-H tumors, high PD-L1 expression correlates with favorable prognosis and strong responses to PD-1/PD-L1 blockade. In contrast, PD-L1 upregulation in MSS tumors may signify immune evasion and portend poorer outcomes [192,193]. Patients with high PD-L1 expression in liver metastases have significantly poorer outcomes. A study involving 62 patients reported a 3-year OS rate of 65.5% and a 3-year RFS rate of 34.7% in this group. In contrast, those with low PD-L1 expression had 3-year OS and RFS rates of 92.7% and 83.8%, respectively. While immunotherapy remains largely ineffective in unselected MSS populations, early-phase studies exploring combinatorial regimens—such as PD-1 inhibitors with anti-VEGF or CTLA-4 agents—are showing potential in biomarker-defined subgroups [194,195]. Reflecting these insights, ESMO and NCCN guidelines now recommend testing for POLE/POLD1 mutations in MSS CRC with high tumor mutational burden particularly in right-sided tumors. Although routine PD-L1 testing is currently limited to MSI-H cases, ongoing research may soon broaden its utility in the evolving immunotherapy landscape of CRLM [196,197].

e. Tumor budding, lymphovascular and perineural invasion

Tumor budding, lymphovascular invasion, and perineural invasion are established histopathological markers of aggressive behavior in colorectal cancer. Tumor budding, characterized by isolated tumor cells or small clusters at the invasive front, is associated with poor prognosis in primary colorectal cancer, correlating with increased risk of lymph node metastasis and poor survival outcomes. However, its prognostic value in CRLM remains less clear, with studies suggesting that tumor budding in CRLM may not independently predict survival outcomes [198]. Tumor budding, defined as single cells or small clusters of tumor cells at the invasive front, is strongly associated with poor OS and RFS in primary colorectal cancer. In one study, high-grade tumor budding was linked to a 5-year RFS of 75.6% versus 92.1% in low-grade cases (*p* = 0.001), with corresponding 5-year OS rates of 93.7% vs. 100%, respectively [199,200]. 

Lymphovascular invasion, defined as the presence of tumor cells within lymphatic or blood vessels, is a significant adverse prognostic factor in both primary colorectal cancer and CRLM. In primary colorectal cancer, lymphovascular invasion is associated with higher rates of recurrence and decreased OS. For instance, a meta-analysis reported a hazard ratio (HR) for OS of 2.15 (95% CI 1.72–2.68) in lymphovascular invasion patients [201]. In CRLM, the presence of lymphovascular invasion in the primary tumor has been linked to poorer survival after liver metastasis resection [202]. Perineural invasion, the invasion of tumor cells along nerves, is also a poor prognostic indicator in colorectal cancer. In primary tumor, perineural invasion is associated with increased risk of recurrence and decreased OS [203]. In CRLM, perineural invasion in the primary tumor has been identified as an independent predictor of poor OS, with a HR of 2.36 (95% CI 1.16–4.82) [204]. In patients undergoing liver resection for CRLM, the presence of perineural invasion in the primary tumor is associated with markedly poorer outcomes, with a 5-year OS of only 29.9% and a recurrence rate of 68.2% [205,206]. These findings underscore the importance of assessing these histopathological features in both primary colorectal cancer and CRLM, as they provide valuable prognostic information that can guide treatment decisions and patient management.

f. Histological growth patterns of liver metastases (pushing vs. replacement patterns)

CRLM exhibits distinct histological growth patterns (HGPs) at the tumor-liver interface, which have been associated with prognosis following surgical resection. The three primary HGPs are as follows:

- *Desmoplastic Growth Pattern* (*dHGP*) characterized by a fibrous rim separating tumor cells from the liver parenchyma, often accompanied by dense immune cell infiltration. This pattern is associated with better OS and DFS. For instance, a study reported a median DFS of 22 months in patients with dHGP [207],
- *Replacement Growth Pattern* (*rHGP*): Tumor cells infiltrate and replace hepatocytes with minimal desmoplastic reaction and inflammatory cell infiltration. This pattern is associated with poorer prognosis, with a hazard ratio for OS of 2.15 compared to dHGP [208]. Patients with rHGP exhibit significantly poorer OS compared to those with pHGP. A study involving 217 patients reported a median OS of 22.8 months for rHGP, compared to 44.2 months for pHGP. Cox regression analysis indicated that the hazard ratio (HR) for death in rHGP patients was approximately 2.5 times higher than in pHGP patients (HR: 0.41, 95% CI: 0.22–0.75, *p* = 0.004). Regarding RFS, rHGP is also associated with worse outcomes. In a cohort of 110 patients undergoing liver resection for CRLM, the DFS rate was significantly lower in patients with rHGP compared to those with pHGP (20.2% vs. 40.5%, *p* = 0.05) [209].
- *Pushing Growth Pattern* (*pHGP*): Tumor cells push aside liver parenchyma without invading hepatocytes, leading to compression without a desmoplastic rim. This pattern is less common but also associated with poor prognosis, with a hazard ratio for DFS of 5.4 compared to dHGP [210,211] (Table 3).

### 3.4. Emerging Prognostic Factors from Precision Medicine

- Circulating tumor DNA (ctDNA): detection of minimal residual disease, early recurrence prediction

Circulating tumor DNA (ctDNA) has emerged as a highly sensitive biomarker for detecting minimal residual disease and predicting early recurrence in colorectal cancer, including patients with CRLM. Numerous studies have demonstrated that ctDNA can detect minimal residual disease in patients even when traditional imaging methods show no evidence of disease [212]. In contrast, ctDNA offers a molecular-level insight, detecting tumor-derived DNA fragments shed into the bloodstream from residual cancer cells even when imaging is negative.

For instance, a study by Tie et al. (2016) found that ctDNA positivity after surgery for CRC was associated with a significantly higher risk of recurrence, with a RFS rate of just 29% for ctDNA-positive patients, compared to 81% for ctDNA-negative patients [213]. This finding highlights ctDNA as an effective biomarker for minimal residual disease that standard imaging cannot yet identify.

Additionally, ctDNA has been shown to provide early detection of recurrence. A study in patients with CRLM who underwent liver metastasectomy revealed that ctDNA was detectable in 50–70% of cases at 6–12 months post-resection, even before clinical or radiological signs of recurrence [214]. Early ctDNA detection following surgery has been linked to a high risk of early recurrence, with 50–60% of ctDNA-positive patients relapsing within 6–12 months. In contrast, only 10–20% of ctDNA-negative patients showed recurrence within the same period. This highlights ctDNA’s potential in guiding postoperative surveillance and tailoring adjuvant therapy for high-risk patients [215,216]. These data underscore the utility of ctDNA as a lead indicator of recurrence, enabling oncologists to identify high-risk patients who may benefit from intensified surveillance or earlier initiation of adjuvant therapies.

Furthermore, serial ctDNA monitoring during systemic therapy or after liver resection provides real-time insights into treatment efficacy and resistance development. The OS for patients who remained ctDNA-negative during follow-up was significantly better, with 5-year OS rates of 80% compared to 50% in ctDNA-positive patients. These findings suggest that ctDNA analysis can be a valuable tool for early intervention, guiding adjustments in therapeutic strategies, such as the initiation of second-line therapies or targeted treatments, to prevent relapse and improve survival outcomes. Incorporating ctDNA analysis into clinical practice could significantly enhance the personalization of treatment in CRLM, helping to identify high-risk patients earlier and enabling tailored interventions that could improve both DFS and OS in the postoperative setting [217,218]. By detecting recurrence months before imaging, ctDNA enables earlier therapeutic interventions that have the potential to improve disease-free survival (DFS) and overall survival (OS). Future clinical trials are warranted to validate ctDNA-guided treatment algorithms, optimize timing and frequency of ctDNA testing, and explore the integration of ctDNA with other biomarkers for enhanced predictive accuracy (Figure S2 in Supplementary Materials).

b. Radiomics: role of quantitative imaging analysis in risk prediction

**Figure 2:**
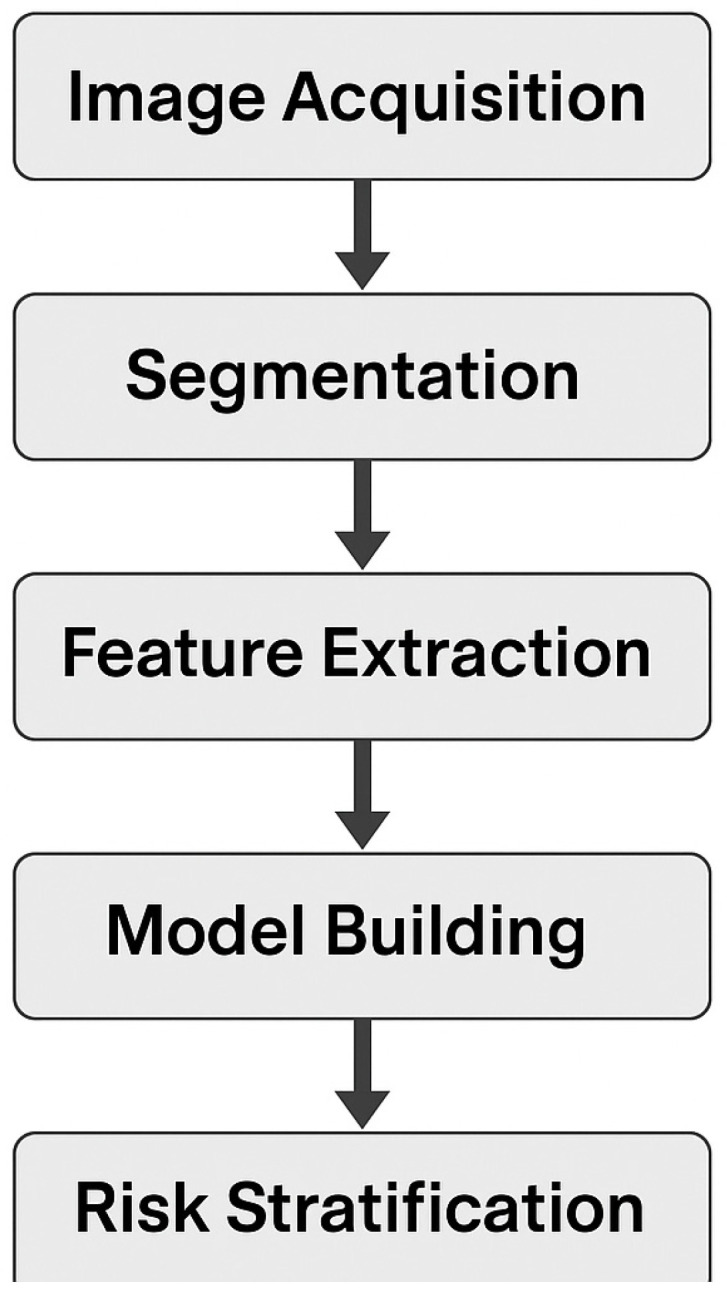
The radiomics workflow.

Radiomics, the extraction of quantitative features from medical images, has emerged as a promising tool for assessing tumor heterogeneity and predicting clinical outcomes in CRLM. By analyzing texture, shape, and intensity patterns in imaging modalities such as computed tomography (CT) and magnetic resonance imaging, radiomics can provide insights into tumor biology that are not apparent through conventional imaging. Recent studies have demonstrated the potential of radiomics in predicting survival and recurrence in CRLM patients. For instance, a study involving 197 patients with CRLM identified several radiomic features associated with OS [219]. Notably, features such as sphericity, correlation, and entropy were found to be statistically significant, with hazard ratios indicating their prognostic value. These findings suggest that radiomic analysis can serve as a non-invasive method to stratify patients based on their risk of poor outcomes [220]. Furthermore, radiomics has been applied to assess the risk of recurrence following treatments like microwave ablation. A study involving 318 patients with recurrent CRLM demonstrated that radiomic features extracted from pre-ablation CT images could predict local tumor progression [221]. The constructed radiomics nomogram showed high predictive performance, with a concordance index (C-index) of 0.912 in the training cohort and 0.89 in the validation cohort. Patients identified as high-risk for local tumor progression had significantly lower 3-year progression-free survival rates compared to low-risk patients, underscoring the utility of radiomics in guiding therapeutic decisions [222]. Despite these promising results, the clinical implementation of radiomics faces challenges such as data standardization, reproducibility, and generalizability. Variability in imaging protocols and analysis methods can impact the consistency of radiomic features across different institutions and platforms. Addressing these issues through standardized imaging protocols and validation studies is crucial for the widespread adoption of radiomics in clinical practice [223] (Figure 2).

c. Gut microbiome: influence on immune response and tumor progression

The gut microbiome plays a crucial role in modulating immune responses and tumor progression in colorectal cancer, including the development of CRLM. Recent studies have shown that specific gut bacteria can influence the tumor microenvironment and treatment responses. For instance, the presence of *Fusobacterium nucleatum* within the tumor has been linked to enhanced PD-L1 expression and an increased immune response to PD-1 checkpoint inhibitors, suggesting a dual role of this bacterium as both a pathogen and a potential enhancer of immunotherapy [224]. Additionally, dysbiosis in the gut microbiome, such as an overabundance of *Desulfovibrio* in patients with CRLM, has been associated with an inflammatory environment both in the gut and liver, facilitating liver metastasis. These microbial alterations can modulate local and systemic immune responses, influencing both disease progression and therapeutic outcomes [225,226]. Clinical studies have demonstrated that microbiome dysbiosis is linked to a higher incidence of early recurrence and poorer survival outcomes in CRLM patients. For example, an analysis of 114 patients with colorectal cancer found that the presence of *Bacteroides fragilis* was associated with poorer disease-free survival, while *Collinsella aerofaciens* was associated with a worse systemic prognostic score, indicating a less favorable tumor microenvironment [227,228]. These findings suggest that gut microbiome composition can significantly impact treatment responses and prognosis in CRLM patients. As such, gut microbiome analysis may emerge as a complementary diagnostic and prognostic tool, offering personalized treatment strategies to improve clinical outcomes (Table 4).

## 4. Discussion

The prognostic assessment of CRLM remains a dynamic and multifaceted challenge. Although numerous prognostic factors have been identified, including tumor burden, molecular alterations, inflammatory markers, and imaging characteristics, the current body of evidence is limited by heterogeneity in study design, endpoints, and patient selection. Most available studies are retrospective, often monocentric, and vary in their definitions of key variables such as resectability and recurrence, thereby limiting their generalizability. The lack of standardization in data collection and analysis underscores the urgent need for prospective validation of proposed prognostic models across diverse populations and treatment settings (Figure S3 in Supplementary Materials).

1. In this complex landscape, no single prognostic factor can adequately capture the full spectrum of recurrence risk or survival potential. Integrating diverse factors—clinical (e.g., number and size of metastases, ASA score), biological (e.g., CEA dynamics, nutritional status), molecular (e.g., RAS/BRAF mutations, MSI, HER2, POLE/POLD1), and imaging-based (e.g., radiomic features, anatomical distribution)—can substantially enhance predictive accuracy [229,230]. This combinatorial approach is not only more reflective of the biological reality of CRLM but also more actionable in a multidisciplinary context. For instance, early postoperative CEA elevation combined with ctDNA positivity and non-desmoplastic histologic growth patterns may identify patients at very high risk of recurrence, warranting intensified surveillance and systemic therapy. Conversely, the absence of high-risk features across domains may justify a de-escalation strategy in selected cases [231,232].

2. These observations support the transition toward a personalized medicine paradigm. Advances in liquid biopsy, radiomics, and multi-omic profiling now allow for dynamic and individualized risk stratification. Circulating tumor DNA enables early detection of minimal residual disease and can precede radiological recurrence by months. Radiomic analysis of preoperative imaging can non-invasively characterize tumor phenotype and aggressiveness. Combined with clinicopathological and molecular data, these modalities are poised to inform tailored therapeutic sequencing, intensity of surveillance, and patient counseling [233,234].

3. Artificial intelligence (AI) represents a major frontier in this evolution, offering the ability to process and integrate complex, high-dimensional datasets into clinically relevant predictions. Recent studies have demonstrated the superiority of AI-driven models, particularly those based on machine learning and deep learning, in predicting survival and recurrence in patients with CRLM. For instance, a model using gradient-boosted trees trained on over 1000 CRLM patients achieved an AUC of 0.773 for OS and 0.635 for recurrence, significantly outperforming traditional statistical models. Another study utilizing gradient-boosted decision trees identified a high-risk subgroup—20% of patients—with a median OS of only 23 months compared to 52 months in the low-risk group (*p* = 0.005), reinforcing the clinical utility of such algorithms [235]. A multi-center effort further validated a random forest model predicting progression-free and OS at 1, 3, and 5 years, with AUCs consistently above 0.70 in both internal and external cohorts [236].

These models demonstrate that AI can move beyond descriptive risk assessment to enable individualized prognostic forecasting. Importantly, their adaptive nature allows real-time updates as new data become available, making them suitable for iterative treatment planning. However, several barriers remain. The interpretability of complex models, integration into clinical workflows, and validation across diverse populations are essential prerequisites for broader implementation. Moreover, AI systems must be embedded within ethical frameworks that ensure transparency, reproducibility, and equity in care delivery [237,238]. In centers that do not have access to a complete multidisciplinary team, AI can serve as a critical tool in bridging the gap, enabling clinicians to evaluate patients and make informed decisions for timely referrals to tertiary centers. By leveraging AI’s capacity for individualized prognostic forecasting, healthcare providers can assess patient risk profiles and predict outcomes with a level of precision that was previously unattainable. These models can be integrated into clinical workflows, allowing for real-time updates and iterative treatment planning. However, successful implementation depends on overcoming barriers such as ensuring the interpretability of complex models, integrating them seamlessly into daily practice, and validating them across diverse patient populations. Additionally, as AI becomes more integrated into clinical decision-making, it must be aligned with ethical frameworks that prioritize transparency, reproducibility, and equitable access to care [239,240]. To translate these innovations into practice, large-scale, prospective, multicenter studies are needed. These should not only validate existing AI models but also evaluate their impact on clinical decision-making, patient outcomes, and healthcare efficiency. International collaboration will be critical to ensure generalizability and to establish standards for data harmonization, model training, and regulatory oversight. Additionally, future clinical trials should incorporate AI-driven stratification strategies to identify patients who may benefit from novel therapies or altered surveillance paradigms [241,242,243]. Despite the promising advances in artificial intelligence (AI) for colorectal liver metastases (CRLM) prognosis and diagnosis, several challenges hinder its widespread clinical adoption. Key obstacles include the “black-box” nature of many AI models, which can limit interpretability and clinician trust. Additionally, regulatory frameworks for AI applications in healthcare remain underdeveloped, creating uncertainty around validation, safety, and ethical considerations. Addressing these barriers through transparent model development, rigorous prospective validation, and clear regulatory guidance will be essential for the successful integration of AI tools into routine CRLM management [244].

## 5. Conclusions

The prognosis of patients with CRLM is shaped by a complex interplay of clinical, biological, molecular, and imaging factors. While traditional indicators such as tumor burden and mutational status remain essential, their predictive power increases markedly when integrated into composite risk models. The convergence of serum biomarkers (e.g., CEA dynamics), molecular alterations (e.g., RAS, BRAF, MSI, HER2, POLE/POLD1), ctDNA-based minimal residual disease detection, and quantitative imaging (radiomics) offers an unprecedented opportunity to personalize both treatment and surveillance strategies. Artificial intelligence is poised to play a transformative role in this paradigm shift. By synthesizing high-dimensional data from multiple sources, AI-based decision-support tools can provide individualized risk forecasts and guide therapeutic sequencing with greater precision than conventional approaches. Recent machine learning models have demonstrated strong prognostic performance, validating their potential for clinical integration.

However, the successful adoption of these tools will require prospective validation, model interpretability, and seamless integration into multidisciplinary workflows. A global effort toward data harmonization, multi-institutional collaboration, and regulatory guidance is now essential. In this evolving landscape, personalized prognostication using AI-enhanced, multimodal algorithms has the potential to refine risk-adapted care, reduce recurrence, and improve long-term survival in patients with CRLM.

## Figures and Tables

**Table 1:** Clinical prognostic factors in CRLM.

FACTOR	IMPACT ON PROGNOSIS	MANAGEMENT IMPLICATIONS	KEY REFERENCES
**GENDER**	Women: 23% less likely to undergo surgery; better OS (40–60 vs. 35–50 months in men)	Consider hormonal factors and tailored chemotherapy dosing (e.g., oxaliplatin adjustments).	[10,11,12,13,14,15]
**AGE**	Elderly (≥65): OS 30–50 months vs. 40–60 months in younger patients	Use geriatric assessments (G8, CGA) to guide treatment intensity.	[16,17,18,19,20]
**ASA SCORE ≥3**	Higher perioperative complications; OS drops to 36 months with major vascular invasion	Prehabilitation, minimally invasive surgery, and ERAS protocols recommended.	[27,28,29,30,31,32]
**NUMBER OF METASTASES**	1–3 lesions: 5-year OS 50–60%; ≥4 lesions: DFS drops to 2.5 months	Neoadjuvant chemotherapy (FOLFOX/FOLFIRI ± biologics) to downsize tumors.	[36,37,38,39,40,41]
**SIZE OF METASTASES**	<5 cm: OS 60 months; ≥5 cm: OS 40 months	Two-stage hepatectomy or ALPPS for large lesions.	[46,47,48,49,50]
**ANATOMIC DISTRIBUTION**	Unilateral: 5-year OS > 50%; bilateral: higher recurrence risk	Portal vein embolization or ALPPS for bilobar disease.	[54,55,56,57,58,59]
**EXTRAHEPATIC METS**	Lung (solitary: 5-year OS 71%; multiple: 48%); Peritoneal (PCI < 10: 5-year OS 53%)	Aggressive local therapy (resection/SBRT) for oligometastatic disease.	[63,64,65,66,67,68,69,70,71,72,73,74,75]

**Table 2:** Strategies to optimize resectability in CRLM.

STRATEGY	DESCRIPTION	OUTCOMES	KEY CONSIDERATIONS
**PORTAL VEIN EMBOLIZATION**	Induces FLR hypertrophy (10–20% growth in 3–8 weeks).	5-year OS 35–45%; 20–30% dropout due to progression.	Low morbidity (2–5%).
**ALPPS**	Rapid FLR growth (70–80% in 7–10 days).	Higher morbidity (40%); mortality 5–10%.	Reserved for selected patients.
**NEOADJUVANT CHEMOTHERAPY**	FOLFOX/FOLFIRI ± biologics; resectability in 30–40% of initially unresectable.	Bevacizumab requires 6-week washout; anti-EGFR for RAS WT.	Monitor for liver injury (sinusoidal obstruction).

**Table 3:** Molecular prognostic factors in CRLM.

BIOMARKER	PREVALENCE	PROGNOSTIC IMPACT	THERAPEUTIC IMPLICATIONS	KEY REFERENCES
**RAS MUTATIONS**	35–45%	Median OS 55.3 months; resistance to anti-EGFR therapies	Bevacizumab preferred over cetuximab.	[165,166,167,168,169]
**BRAF V600E**	1–6.1%	Median OS 16.2 months; aggressive biology	BRAF/MEK inhibitors (e.g., encorafenib + binimetinib).	[167,168,169,170,171,172,173]
**MSI-H**	5–15%	Favorable with immunotherapy (HR 0.57 for OS vs. chemotherapy)	Pembrolizumab/nivolumab in first-line.	[174,175,176,177,178,179,180,181]
**HER2 AMPLIFICATION**	3–5% (KRAS WT)	Resistance to anti-EGFR; response to trastuzumab + pertuzumab (30–50% response rate)	Dual HER2 blockade or trastuzumab deruxtecan.	[182,183,184,185,186,187,188,189]
**POLE/POLD1**	1–2%	Hypermutated phenotype; favorable response to immunotherapy	Checkpoint inhibitors (even in MSS tumors).	[190,191,192,193,194,195,196,197]

**Table 4:** Emerging prognostic tools in CRLM.

TOOL	APPLICATION	CLINICAL UTILITY	LIMITATIONS	KEY REFERENCES
**CTDNA**	Detects minimal residual disease; predicts recurrence (50–60% relapse if +postop)	Guides adjuvant therapy decisions; early intervention for high-risk patients.	Standardization and cost.	[212,213,214,215,216,217,218]
**RADIOMICS**	Quantifies tumor heterogeneity (e.g., sphericity, entropy)	Predicts local progression post-ablation (AUC 0.89–0.91).	Reproducibility across imaging protocols.	[219,220,221,222,223]
**GUT MICROBIOME**	*Fusobacterium nucleatum* linked to PD-L1 expression; *B. fragilis* worsens DFS	Potential for microbiome modulation (e.g., probiotics) to enhance immunotherapy.	Causality not established.	[224,225,226,227,228]

## Data Availability

No new data were created.

## Supplementary Materials

The following supporting information can be downloaded at: https://www.mdpi.com/article/10.3390/cancers17152539/s1, Figure S1: Colorectal cancer (CRC) pathogenesis and molecular pathways, showcasing key drivers alongside clinical markers; Figure S2: Clinical, molecular, and emerging biomarkers in CRC; Figure S3: The impact of prognostic factors on the decisional algorithm in CRLM. References [245,246,247,248,249,250] are cited in the Supplementary Materials.
