# Supplementary material for: Prognostic Factors in Colorectal Liver Metastases: An Exhaustive Review of the Literature and Future Prospectives
Source: Cancers (Basel). 2025 Jul 31;17(15):2539. doi: 10.3390/cancers17152539 (PMC12346680; doi:10.3390/cancers17152539)
Supplement: Supplementary file 1 [file cancers-17-02539-s001.zip › cancers-3722592-supplementary.pdf]

cancers

Review

Prognostic Factors in Colorectal Liver Metastases: An Exhaustive Review of the Literature and Future Prospectives

Maria Conticchio [1,*], Emilie Uldry [1,2], Martin Hübner [1,2], Antonia Digklia [1,3], Montserrat Fraga [1,4], Christine Sempoux [1,5], Jean Louis Raisaro [1] and David Fuks [1,2]

[1] Faculty of Biology and Medicine, University of Lausanne, 1005 Lausanne, Switzerland; emilie.uldry@chuv.ch (E.U.); martin.hubner@chuv.ch (M.H.); antonia.digklia@chuv.ch (A.D.); montserrat.fraga@chuv.ch (M.F.); christine.sempoux@chuv.ch (C.S.); jeanlouis.raisaro@unil.ch (J.-L.R.); david.fuks@chuv.ch (D.F.)
[2] Department of Visceral Surgery, Centre Hospitalier Universitaire Vaudois (CHUV), 1005 Lausanne, Switzerland
[3] Department of Oncology, Centre Hospitalier Universitaire Vaudois (CHUV), 1005 Lausanne, Switzerland
[4] Department of Gastroenterology, Centre Hospitalier Universitaire Vaudois (CHUV), 1005 Lausanne, Switzerland
[5] Department of Pathology, Centre Hospitalier Universitaire Vaudois (CHUV), 1005 Lausanne, Switzerland
* Correspondence: maria.conticchio@unil.ch; Tel.: +39-3929786255

The impact of socioeconomic status in patients with CRLM

Socioeconomic status (SES) plays a significant role in the outcomes of patients with colorectal liver metastases (CLM). Studies indicate that individuals with lower SES tend to have poorer survival rates and are less likely to undergo potentially curative treatments like liver resection (1).

Patients with lower SES are less likely to be referred for and undergo surgical resection of liver metastases, even when the metastases are considered resectable(2).These disparities may be linked to factors like access to specialized care, adherence to treatment recommendations, and delays in diagnosis or treatment (3).

Lower SES is associated with worse overall survival (OS) following diagnosis and treatment for CLM. This disparity is evident even when comparing outcomes after surgical resection.

Socioeconomic factors significantly influence both the treatment and survival outcomes of patients with colorectal liver metastases. Addressing these disparities through improved access to care, targeted interventions, and awareness campaigns is crucial for ensuring equitable outcomes for all patients with CRLM.

The Charlson Comorbidity Index in patients with CRLM

The Charlson Comorbidity Index (CCI)(4) is widely used to quantify comorbidity burden and predict overall survival in oncology patients, including those with colorectal liver metastases (CRLM). The index assigns weighted scores to chronic conditions, with higher total scores indicating increased mortality risk. Several studies have demonstrated that higher CCI scores correlate with poorer survival outcomes in CRLM cohorts(5). For example, age-adjusted CCI ≥2 was independently associated with reduced overall survival in a large propensity-matched series of CRLM patients, showing a hazard ratio (HR) of approximately 1.25 per comorbidity point . Additionally, a single-center study including older CRLM patients found that elevated CCI significantly predicted postoperative mortality, with a multivariate HR of 5.2 for scores ≥2 . Moreover, CCI demonstrated superior prognostic accuracy over other comorbidity indices in colorectal cancer populations. In recognition of this, several MDT guidelines recommend

 https://doi.org/10.3390/cancers17152539

incorporating comorbidity scoring into preoperative evaluation to optimize risk stratification and individualized treatment planning(6).

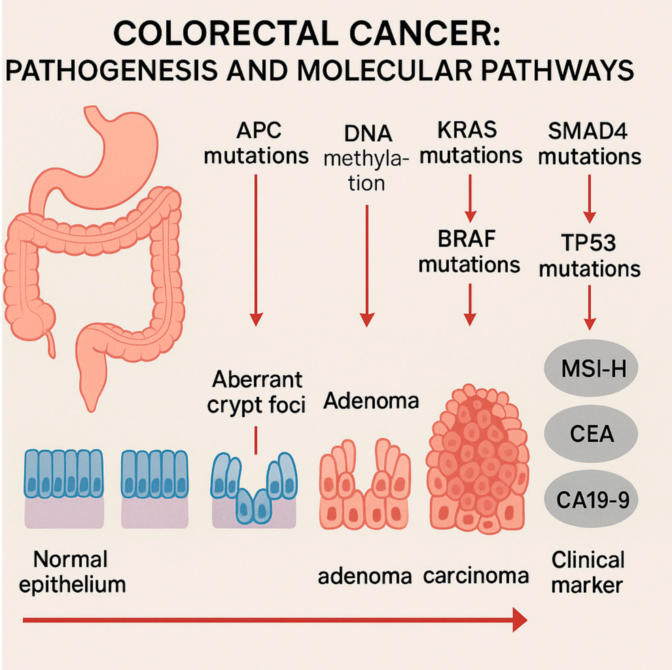

Figure S1. Colorectal cancer (CRC) pathogenesis and molecular pathways, showcasing key drivers alongside clinical markers.

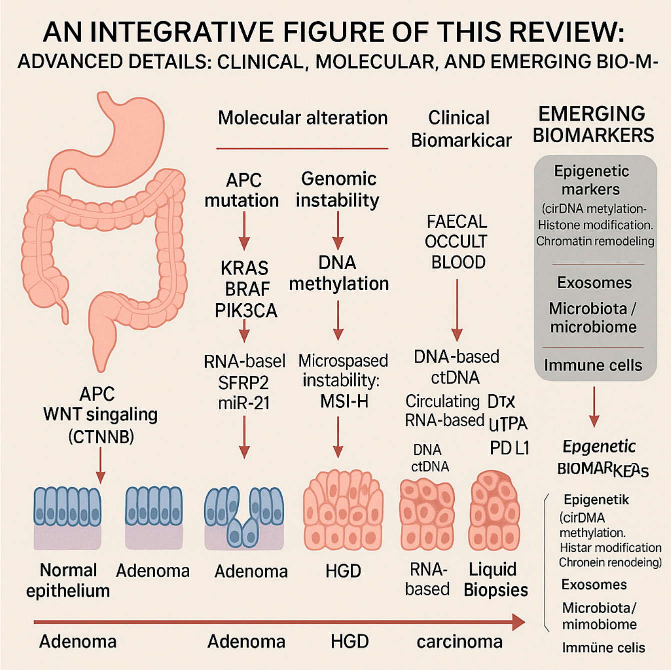

Figure S2. Clinical, molecular, and emerging biomarkers in CRC.

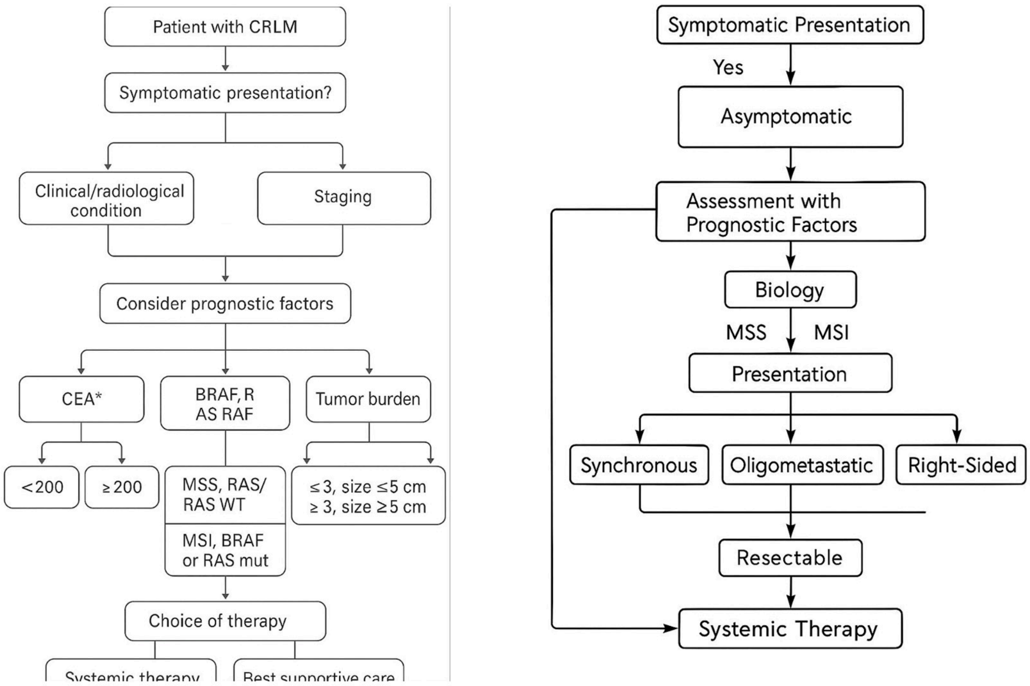

Figure S3. The impact of prognostic factors on the decisional algorithm in CRLM.

References

1. Rieser CJ, Hoehn RS, Zenati M, Hall LB, Kang E, Zureikat AH, Lee A, Ongchin M, Holtzman MP, Pingpank JF, Bartlett DL, Choudry MHA. Impact of Socioeconomic Status on Presentation and Outcomes in Colorectal Peritoneal Metastases Following Cytoreduction and Chemoperfusion: Persistent Inequalities in Outcomes at a High-Volume Center. Ann Surg Oncol. 2021 Jul;28(7):3522-3531. doi: 10.1245/s10434-021-09627-2. Epub 2021 Mar 9. Erratum in: Ann Surg Oncol. 2021 Dec;28(Suppl 3):875. doi: 10.1245/s10434-021-10045-7. PMID: 33687614; PMCID: PMC8184539.
2. Sell NM, Shafique N, Lee H, Lee GC, Tanabe KK, Ferrone CR, Blaszkowsky LS, Hong TS, Wo J, Qadan M. Socioeconomic determinants of the surgical treatment of colorectal liver metastases. Am J Surg. 2020 Oct;220(4):952-957. doi: 10.1016/j.amjsurg.2020.02.019. Epub 2020 Feb 21. PMID: 32107013.
3. Vallance AE, van der Meulen J, Kuryba A, Braun M, Jayne DG, Hill J, Cameron IC, Walker K. Socioeconomic differences in selection for liver resection in metastatic colorectal cancer and the impact on survival. Eur J Surg Oncol. 2018 Oct;44(10):1588-1594. doi: 10.1016/j.ejso.2018.05.024. Epub 2018 May 26. PMID: 29895508.
4. Charlson ME, Pompei P, Ales KL, MacKenzie CR. A new method of classifying prognostic comorbidity in longitudinal studies: development and validation. J Chronic Dis. 1987;40(5):373-83. doi: 10.1016/0021-9681(87)90171-8. PMID: 3558716.
5. Zhang, X., Wang, X., Wang, M., Gu, J., Guo, H., Yang, Y., … Li, Q. (2022). Effect of comorbidity assessed by the Charlson Comorbidity Index on the length of stay, costs, and mortality among colorectal cancer patients undergoing colorectal surgery. Current Medical Research and Opinion, 39(2), 187–195. https://doi.org/10.1080/03007995.2022.2139053
6. Lam CSN, Bharwani AA, Chan EHY, Chan VHY, Au HLH, Ho MK, Rashed S, Kwong BMH, Fang W, Ma KW, Lo CM, Cheung TT. A machine learning model for colorectal liver metastasis post-hepatectomy prognostications. Hepatobiliary Surg Nutr. 2023 Aug 1;12(4):495-506. doi: 10.21037/hbsn-21-453. Epub 2022 Jul 12. PMID: 37601005; PMCID: PMC10432293.

Disclaimer/Publisher's Note: The statements, opinions and data contained in all publications are solely those of the individual author(s) and contributor(s) and not of MDPI and/or the editor(s). MDPI and/or the editor(s) disclaim responsibility for any injury to people or property resulting from any ideas, methods, instructions or products referred to in the content.
